# Successful Hysteroscopic Resection of Caesarean Scar Ectopic Pregnancy With Subsequent Intrauterine Pregnancy: A Case Report

**DOI:** 10.7759/cureus.91291

**Published:** 2025-08-30

**Authors:** Nahla Rashad Hamdan Abdelrahman, Marwa M Elboghdady, Susan S Khalafallah, Wael Hosni

**Affiliations:** 1 Obstetrics and Gynaecology, HMS Al Garhoud Hospital, Dubai, ARE; 2 Gynaecology, HMS Al Garhoud Hospital, Dubai, ARE; 3 Anaesthesiology, HMS Al Garhoud Hospital, Dubai, ARE

**Keywords:** bipolar diathermy, caesarian scar, ectopic pregnancy, niche or scar pregnancy, operative hysteroscopy

## Abstract

A 36-year-old woman (G5P3A1) with two previous lower-segment caesarean sections presented with a positive pregnancy test and minimal vaginal bleeding. Transvaginal ultrasound demonstrated a six-week gestational sac implanted within the prior scar; initial serum beta human chorionic gonadotropin (β-hCG) was 7775 mIU/mL. After counselling, she elected fertility-preserving hysteroscopic resection. β-hCG level reached 24000 mIU/mL, operative hysteroscopy with bipolar loop excision of the gestational sac was performed uneventfully. β-hCG fell to 6095 mIU/mL on postoperative day one and was undetectable by week four. Six-week ultrasound confirmed complete scar resolution. Five months later the patient conceived spontaneously and progressed to a viable intrauterine pregnancy.

## Introduction

Caesarean scar ectopic pregnancy (CSP) represents a rare but hazardous form of ectopic pregnancy in which the embryo implants in a previous caesarean section scar [1]. Recently updated international recommendations have refined diagnostic criteria and management algorithms [2]. Population-based data estimate its incidence at approximately one in 1,800-2,200 pregnancies, mirroring the global increase in caesarean section rates [3,4]. CSP constitutes up to 6 % of all ectopic pregnancies in women with a prior caesarean delivery [5].

Documented risk factors include multiple caesarean sections, other uterine surgery, assisted reproductive techniques and short inter-pregnancy intervals [6,7]. Diagnosis is primarily sonographic, demonstrating an empty uterine cavity and endocervical canal with a gestational sac embedded in the anterior lower uterine segment myometrium at the isthmic level [8]. Magnetic resonance imaging (MRI) adds value when sonographic findings are equivocal, delineating myometrial thickness and bladder involvement [9].

If untreated, CSP can progress to uterine rupture, catastrophic haemorrhage and loss of fertility [1,10]. Management options include systemic or local medical therapy, hysteroscopic, laparoscopic or open surgical resection, and interventional radiology procedures such as uterine artery embolisation [11-13]. Selection depends on gestational age, viability, beta human chorionic gonadotropin (β-hCG) level and future fertility wishes.

We present a successful hysteroscopic resection of CSP followed by spontaneous intrauterine pregnancy, underscoring hysteroscopy as a viable fertility-preserving approach.

## Case presentation

A 36-year-old woman (gravida 5, para 3, abortus 1) attended our clinic after a positive urine pregnancy test. She reported mild bloodstained vaginal discharge without abdominal pain. Her history included two lower-segment caesarean sections; otherwise, unremarkable. On abdominal examination, there was no abdominal tenderness, rigidity, or rebound. Vaginal examination revealed mild spotting. 

Initial serum βhCG was 7,775 mIU/mL. Transvaginal ultrasound revealed a six-week gestational sac embedded in the anterior lower uterine segment at the caesarean scar site, with no intrauterine or adnexal pregnancy sac, confirming CSP [8].

The management plan was admitting the patient, using a minimal invasive surgical approach like hysteroscopy to remove the ectopic gestational sac completely under general anaesthesia and to be discharged from the hospital the next day. After counselling, hysteroscopic resection was chosen in view of the patient’s fertility wishes and stable condition [11].

**Video 1:** Case Presentation on Scar Ectopic Pregnancy

By the procedure date, βhCG had risen to 24,000 mIU/mL. Under general anaesthesia, the gestational mass was completely resected with a bipolar hysteroscopic resectoscope; haemostasis was achieved, and no complications occurred.

Postoperatively, βhCG fell to 6,095 mIU/mL on day one, and the patient was discharged home. βhCG was measured again and became undetectable by week four. Ultrasound at six weeks showed complete scar resolution.

Five months later, the patient conceived spontaneously. Transvaginal ultrasound demonstrated a single intrauterine gestational sac with a crown-rump length consistent with six weeks and five days and a pulsating foetal pole, indicating a viable intrauterine pregnancy. 

## Discussion

Although CSP accounts for a small percentage of all ectopic pregnancies, delayed recognition can lead to severe haemorrhage, uterine rupture, and hysterectomy [1,10,14]. Early diagnosis via high-resolution transvaginal ultrasound is essential and often reveals an empty uterine cavity, an empty endocervical canal, and a gestational sac embedded in the myometrial defect of the anterior uterine wall [8].

Management is guided by gestational age, foetal viability, serum β-hCG concentration, and the patient’s future fertility plans. Medical management with systemic or local methotrexate is considered for early, nonviable pregnancies when βhCG is <5,000 mIU/mL, although failure rates increase above this threshold [15,16].

Surgical intervention is generally selected when the gestational sac is viable, serum βhCG exceeds 5,000 mIU/mL, or initial medical therapy fails [15,16]. Available procedures include ultrasound-guided dilation and curettage, hysteroscopic excision, laparoscopic wedge resection, and open laparotomy [11-13]. Thorough pre-operative assessment-identifying high-risk cases and those suitable for conservative approaches-remains essential to minimise complications [11].

Ideally, surgery should be performed before nine weeks’ gestation to reduce the likelihood of visceral injury. Current evidence recommends tailoring the operative route to CSP subtype: hysteroscopy for endogenic lesions and laparoscopy for exogenic lesions, with combined approaches reserved for select scenarios [17,18]. A contemporary narrative review presents one of the few CSP papers that lays out a patient-tailored decision algorithm decision algorithm reinforcing this strategy; by stratifying pregnancies into endogenic versus exogenic forms and weighing residual-myometrium thickness, sac size, haemodynamic status, and fertility wishes, it advocates hysteroscopic evacuation for contained type I lesions (≥ 3 mm myometrium; sac < 3 cm) and laparoscopic or open resection for type II lesions characterised by a thin myometrial mantle, active bleeding, or the need for simultaneous scar repair. The review therefore exemplifies how explicit, personalised criteria can rationalise the choice between hysteroscopy, laparoscopy, and laparotomy in routine practice [17].

Evidence from a retrospective series of 112 CSPs further supports this schema: the hysteroscopy group recorded a mean operating time of 25 min, blood loss of 22.6 mL, and hospital stay of 5.24 days. The laparoscopy group required 108 min, lost 305.8 mL of blood, and remained hospitalised for 8.65 days; all differences were statistically significant [19]. Similarly, a retrospective single-centre cohort of 314 cases with caesarean scar pregnancy compared pituitrin-assisted hysteroscopic curettage, methotrexate-guided curettage, and laparoscopic or abdominal scar resection. The hysteroscopic approach yielded the least blood loss, shortest hospital admission, and fastest β-hCG and menstrual recovery. Operative success and subsequent pregnancy rates were higher for type I-II lesions managed hysteroscopically, whereas laparoscopy produced fewer complications for type III lesions, again highlighting the value of tailoring the surgical route to ultrasound subtype [20].

Long-term reproductive outcomes also appear encouraging: in a multicentre series of 439 cases, hysteroscopy-based strategies achieved 93.6 % technical success with major complications in 8.2% of procedures. Of the women who subsequently attempted conception, 37 conceived and 22 delivered healthy term infants by scheduled caesarean section, with no uterine ruptures. That cohort also reported a 10.8% recurrence rate of caesarean scar pregnancy [20]. Although prospective data remain limited, these findings support hysteroscopy as a fertility-preserving option when coupled with diligent follow-up and early first-trimester imaging in future pregnancies.

Our patient’s uneventful hysteroscopic resection, rapid β-hCG decline, and subsequent viable intrauterine pregnancy add to this limited but growing body of evidence, further corroborating hysteroscopic management as an effective, fertility-preserving option when applied to appropriately selected cases [20].

## Conclusions

Early, high-resolution transvaginal ultrasound remains the cornerstone of CSP diagnosis, allowing prompt risk stratification and informed management choices. In appropriately selected endogenic lesions, hysteroscopic resection before nine weeks’ gestation combines surgical precision with minimal blood loss, brief hospitalisation, and rapid β-hCG resolution. Our patient’s uncomplicated procedure and subsequent spontaneous intrauterine pregnancy reinforce hysteroscopy as an effective, fertility-preserving option and underscore the importance of personalised algorithms that match operative approach to ultrasound subtype, myometrial thickness, and reproductive goals. Continued accumulation of case-level and prospective data will further refine these selection criteria and optimise outcomes for patients facing this challenging diagnosis.
